# Role of Signaling Molecules Sodium Nitroprusside and Arginine in Alleviating Salt-Induced Oxidative Stress in Wheat

**DOI:** 10.3390/plants11141786

**Published:** 2022-07-06

**Authors:** Marwa M. Ragaey, Mervat Shamoon Sadak, Mona F. A. Dawood, Nermin H. S. Mousa, Rania Samy Hanafy, Arafat Abdel Hamed Abdel Latef

**Affiliations:** 1 Botany and Microbiology Department, Faculty of Science, New Valley University, Al-Kharja 72511, Egypt; 2 Botany Department, Agricultural and Biological Research Institute, National Research Centre, 33 El Bohouth Street, Dokki, Giza 12622, Egypt; mervat_sh24@yahoo.com; 3 Botany and Microbiology Department, Faculty of Science, Assiut University, Assiut 761515, Egypt; mo_fa87@aun.edu.eg (M.F.A.D.); mousanhs@aun.edu.eg (N.H.S.M.); 4 Biological and Geological Sciences Department, Faculty of Education, Ain Shams University, Cairo 11341, Egypt; raniasamy@edu.asu.edu.eg; 5 Botany and Microbiology Department, Faculty of Science, South Valley University, Qena 83523, Egypt

**Keywords:** amino acid profile, antioxidant enzymes, arginine, salt stress, sodium nitroprusside

## Abstract

Nitric oxide (NO) is a well-accepted signaling molecule that has regulatory effects on plants under various stresses. Salinity is a major issue that adversely affects plant growth and productivity. The current study was carried out to investigate changes in the growth, biochemical parameters, and yield of wheat plants in response to NO donors, namely sodium nitroprusside (SNP) (2.5 and 5.0 mM) and arginine (10 and 20 mM), under two salinity levels (1.2 mM and 85.5 mM NaCl). Salinity stress significantly decreased the lengths and weights of plant parts (shoot, tiller, and root) and reduced the flag leaf area, photosynthetic pigments, indole acetic acid (IAA), and yield and its components. Moreover, salt stress induced a significant accumulation of some osmoprotectants (total soluble sugars (TSS) and amino acids, especially proline) and triggered the accumulation of hydrogen peroxide (H_2_O_2_) and lipid peroxidation in wheat leaves. In contrast, arginine and SNP treatments significantly mitigated the negative impacts of salinity on growth and productivity via enhancing photosynthetic pigments, nitrate reductase, phenolic compounds, IAA, TSS, free amino acids, and proline. In addition, SNP and arginine potentially reduced oxidative damage by decreasing H_2_O_2_ and lipid peroxidation through the induction of antioxidant enzymes. The individual amino acid composition of wheat grains under the interactive effect of salinity and NO sources has been scarcely documented until now. In this study, the NO sources restrained the reduction in essential amino acids (isoleucine and lysine) of wheat grains under salinity stress and further stimulated the contents of non-essential and total aromatic amino acids. Interestingly, the applied protectants recovered the decrease in arginine and serine induced by salinity stress. Thus, SNP or arginine at the levels of 5.0 and 20 mM, respectively, had a profound effect on modulating the salt stress of wheat throughout the life cycle.

## 1. Introduction

Salinity is a serious global problem that suppresses crop productivity. Salt stress affects arid and semi-arid regions in the world, causing significant harm to crops. Salinity obstructs the redox homeostasis of the cell and causes a wide range of adverse effects on plant growth and metabolism. Excess salt in the soil prompts ion toxicity, such as that from sodium Na^+^ and chloride Cl^−^, and dehydration/osmotic stress, which deteriorates the structure and function of proteins, lipids, and DNA, causing biomembrane dysfunction and severely affecting tissues and organelles [1,2]. Thus, major adverse alterations have appeared in phenological traits and physiological pathways, including water uptake, water use efficiency, nutrient uptake, osmoregulation, transpiration, respiration, stomatal closure, photosynthesis, and the assimilation of photosynthates [3]. This attenuation of photosynthesis and stomatal closure by salinity energizes chloroplasts via disproportionate excitation energy, which stimulates the production of excessive reactive oxygen species (ROS) that disturb plant cell homeostasis [2]. In this regard, ROS mainly cause damage to the cellular membrane by inducing lipid peroxidation; they can harm DNA, proteins, and chlorophyll [4]. In contrast, the cell produces an antioxidative system, which is the major definitive protection against oxidative damage. The antioxidative system is composed of several antioxidant enzymes such as superoxide dismutase (SOD), catalase (CAT), ascorbate peroxidase (APX), non-enzymatic antioxidants, and secondary metabolites [5]. A further strategy to restrain the adverse effects of salinity is osmoregulation which is connected to various osmoprotectant compounds such as soluble sugars, proteins, and amino acids [6,7]. Amino acids are the major structural and functional building units in the cell. They are also connected to molecular plant defensive processes involved in free radical scavenging and/or production, gene regulation, and defensive product creation [8]. As has been reviewed recently by [9], amino acids are considered important precursors for the production of secondary metabolites and signaling compounds such as polyamines, plant hormones, immune signaling compounds, and metabolites with biological functions and health-promoting properties. Therefore, studying the changes in the amino acid profile is of interest in the field of salinity stress as a base for various important physiological pathways in the plant cell. 

Environmental or abiotic stress drives the development of several mechanisms through which plant cells may perceive environmental stimuli or signals and respond in ways that assist plants to cope with these adverse situations [10]. The detection of biotic or abiotic stress triggers signaling cascades that stimulate the synthesis of signaling molecules such as reactive oxygen species (ROS), calcium (Ca^+2^), and nitric oxide (NO) [11]. 

The involvement of NO in salinity tolerance has drawn much attention in the past decade. The enhancement of endogenous NO level, as well as its exogenous application, has been found to mitigate salinity stress through multiple mechanisms. Sodium nitroprusside (SNP) is a widely used NO donor; it is a diffusible bioactive signaling molecule that plays a crucial role in plants’ growth, metabolism processes, and productivity under normal or stressed conditions [12,13]. Another important source of NO is arginine, which is an important amino acid related to the production of polyamines, proline, or nitric oxide [14]. Thus, arginine improves many biological activities in plants by participating in cellular defense against oxidative damage, lowering lipid peroxidation, and scavenging free radicals [15]. The exogenous application of such signaling compounds could trigger a defense response against the encountered stress. NO influences signal transduction and responses to several biotic and abiotic stresses [16] by positively affecting plant health, development, and metabolic pathways [17]. Recently, the exogenous application of NO was shown to improve phenolics, flavonoids, glutathione metabolism, ascorbic acid, soluble sugars, proline, and protein in plants. The application of NO decreases the production of free radicals and lowers lipid peroxidation. NO treatment enhances photosynthesis, stomatal conductance, transpiration rate, photosystem efficiency, relative water content, chlorophyll content, and nutrient uptake in plants under abiotic stress conditions [10]. Few studies have documented the role of the foliar application of NO donors or precursors in amino acid pathways of salinized plants. 

Wheat (*Triticum aestivum*) is considered the most important grain-producing cereal and the first crop among cereals consumed by humans [18]. Wheat yield might drop by 6% because of stressful environments, according to several climate models [19]. Soil salinization has the most negative impact on wheat productivity and quality via modifying physiological and biochemical processes in plants. Most previous studies concentrated on the effects of salt on grain production [20], but the link between salt tolerance and grain quality is poorly understood, especially under the use of protecting agents. Therefore, this study was conducted to investigate the effects of foliar application of arginine and SNP on the alleviation of the morpho-physiological changes in wheat plants subjected to salinity in terms of growth, oxidative damage, membrane stability, osmoprotectants, hormonal imbalance, and the antioxidant system. As there are limitations to the study of the physiology of grains under salinity and/or NO applications, detecting the changes in grains’ content of individual amino acids and sugars could add a benefit to the whole change in the metabolism under the studied conditions. 

## 2. Results

### 2.1. Effect of SNP or Arginine on the Growth Indices of Wheat Plants Grown under Salinity Stress

The data presented in Table 1 show that the irrigation of wheat plants with saline water (85.5 mM NaCl) caused significant decreases in the growth indices of wheat compared with the control plants (S_0_; 1.2 mM NaCl). In this regard, the shoot length was reduced from 56.3 to 43.7 cm, recording a reduction of 22.49% compared to the control. Additionally, the tiller-associated traits, namely number of leaves/tillers, flag leaf area, tiller fresh weight, and tiller dry weight, were attenuated from 4.7, 30.5, 5.6, and 1.8 (control plants) to 4.3, 24.2, 4.1, and 1.1 (stressed plants), with reductions of 7.14%, 20.56%, 27.55%, and 40.34%, respectively. Furthermore, the roots of *wheat* plants were negatively affected by salinity stress, where root length, root fresh weight, and root dry weight were reduced from 13.7, 1.8, and 0.92 (control) to 11.7, 1.1, and 8.4 (stressed plants), with a percent reduction of 17.14%, 27.9%, and 10.32%, respectively. *In contrast*, different treatments of either arginine (10 and 20 mM) or SNP (2.5 and 5.0 mM) increased various growth parameters studied under both normal and salinized conditions. Additionally, the data clearly show that the higher concentration of arginine or SNP was more effective than the lower one. The highest values of growth parameters were obtained at 5.0 mM SNP followed by 20 mM arginine under normal irrigation conditions and salt stress conditions (Table 1). The level of 20 mM arginine was the most effective treatment, followed by 5.0 mM SNP, except for the shoot length, under both normal and salinity stress conditions, and the number of leaves/tillers and flag leaf area under normal conditions. 

### 2.2. Effect of SNP or Arginine on the Photosynthetic Pigments of Wheat Plants Grown under Salinity Stress

**Figure 1:**
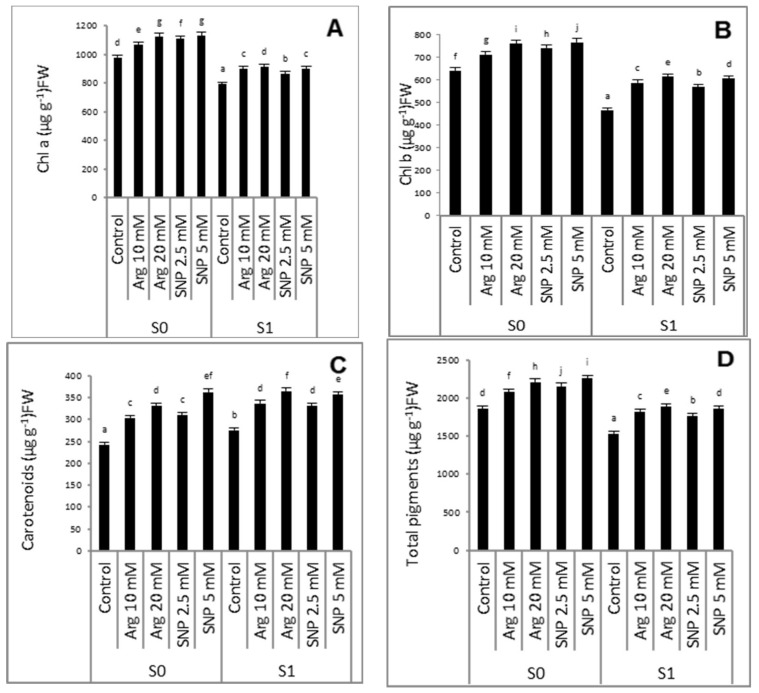

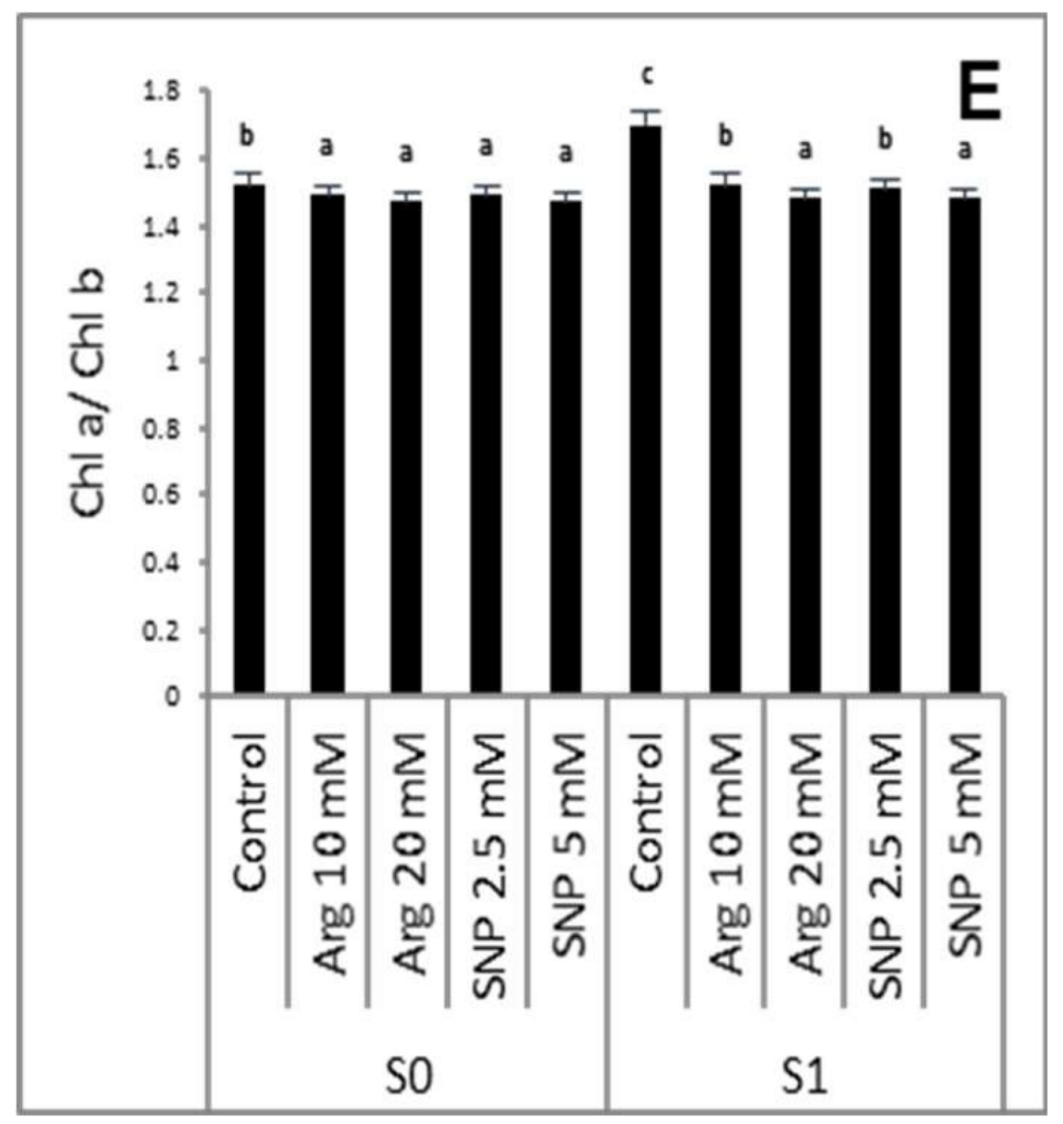
Effect of arginine or SNP on photosynthetic pigments indices of wheat plants (chlorophyll a (**A**), chlorophyll b (**B**), carotenoids (**C**), and total pigments (**D**), chlorophyll a/chlorophyll b (**E**)) under salinity stress. S_0_ = 1.2 mM NaCl, S_1_ = 85.5 mM NaCl. All the statistical differences were presented relative to the untreated control. Data are means of three replicates ± SE; means with different letters are significantly different (*p* ≤ 0.05).

Irrigation of wheat plants with saline water induced a marked decrease in Chl *a*, Chl *b*, and total pigments (Figure 1A,B,D) relative to the non-salinized plants. However, the carotenoid content and Chl *a*/Chl *b* ratio (Figure 1C,E) were boosted significantly compared to the non-salinized plants. The foliar treatments with either arginine or SNP restrained the reduction in chl *a*, chl *b*, and total pigments as compared with the untreated controls. Further improvement in the carotenoid content was found in leaves that received arginine or SNP under both conditions. The data show that the higher concentrations of arginine or SNP were more effective in increasing the photosynthetic parameters than the lower concentrations. It is worth mentioning that 5.0 mM SNP was the most effective treatment for non-salinized wheat plants, while 20 mM arginine was the most effective treatment under salinity stress (Figure 1).

### 2.3. Effect of SNP or Arginine on the Endogenous Phytohormone IAA and Phenolic Contents of Wheat Plants Grown under Salinity Stress

**Figure 2:**
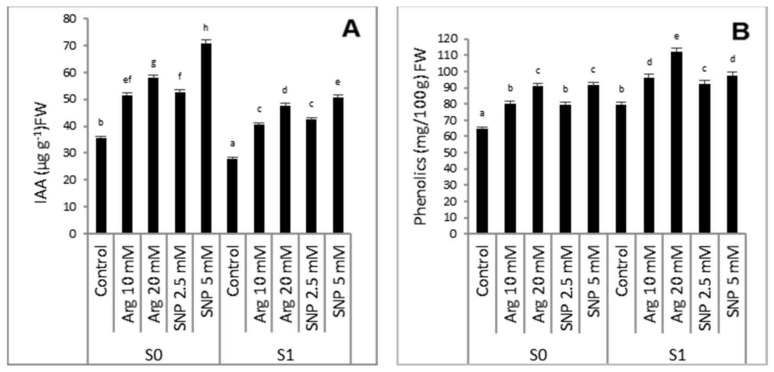
Effect of arginine or SNP on indole acetic acid content (**A**) and phenolic compounds (**B**) of wheat plant under salinity stress. S_0_ = 1.2 mM NaCl, S_1_ = 85.5 mM NaCl. All the statistical differences were presented relative to the untreated control. Data are means of three replicates ± SE; means with different letters are significantly different (*p* ≤ 0.05).

The data in Figure 2 show that exposure of wheat plants to salinity stress significantly decreased the IAA content compared to the control. However, phenolic contents increased significantly in response to salt stress compared to the non-salinized plants. Arginine or SNP not only restricted the reduction in IAA but also increased its content compared to the control, especially 5 mM SNP. Phenolic compounds were enhanced by the applied protectants, and the highest stimulation was found for 20 mM arginine-treated plants grown under saline irrigation.

### 2.4. Effect of SNP or Arginine on the Compatible Solutes of Wheat Plants Grown under Salinity Stress

Salinity stress induced significant increases in the proline, TSS, and free amino acid contents of the wheat plants as compared with the control plants. Moreover, external treatments of arginine or SNP significantly increased the contents of osmoprotectants regardless of the soil condition. Comparatively, the higher concentration of either arginine or SNP was more effective than the lower concentration either under irrigation with normal water or under salinity-stressed conditions (Table 2).

### 2.5. Effect of SNP or Arginine on H_2_O_2_ and Lipid Peroxidation of Wheat Plants Grown under Salinity Stress

The irrigation of wheat plants with saline water significantly increased H_2_O_2_ and lipid peroxidation expressed in the MDA content as compared with those plants irrigated with tap water. However, the external treatments using different concentrations of arginine or SNP decreased the contents of H_2_O_2_ and MDA as compared to their corresponding controls (Table 3). It was noted that greater concentrations of either arginine or SNP had a higher capacity to decrease H_2_O_2_ and lipid peroxidation than the lower concentrations. 

### 2.6. Effect of SNP or Arginine on Antioxidant Enzymes as Well as Nitrate Reductase Activity of Wheat Plants Grown under Salinity Stress 

Regarding the antioxidant enzymes of salinized plants, CAT, SOD, and POX were enhanced relative to control plants (Table 3). In contrast, salinity stress reduced NR activity to 296.83 n mole NO_2_/g fresh weight/h as compared with unstressed control plants (320.76 n mole NO_2_/g fresh weight/h). Moreover, exposing plants to arginine or SNP under normal or saline irrigation conditions caused a further increase in CAT, SOD, and POX and alleviated the reduction in NR activity as compared with their corresponding untreated controls. 

### 2.7. Effect of SNP or Arginine on the Yield and Yield Component Traits of Wheat Plants Grown under Salinity Stress 

The adverse effects of salinity and the mitigation effects of NO donors on yield and morphological traits of harvested plants were assessed. The yield (spike length, spike weight, grain weight, and 1000 kernel weight), morphological traits at harvest (shoot length and shoot weight), and total carbohydrates of wheat plants under salinity stress were reduced drastically as compared with the control plants (Table 4). In addition, there were significant increases in wheat yield components and carbohydrate contents caused by the exposure to either arginine or SNP, with higher concentrations of both materials being more effective.

### 2.8. Effect of SNP or Arginine on Amino Acid Profile of Wheat Grains Grown under Salinity Stress

The highest levels of SNP or arginine, as the best concentrations in alleviating the damage caused by salinity, were used for studying the changes in the amino acid profile of wheat grains. Proline, glutamic acid, lysine, and glycine, in that order, were the most abundant amino acids in the wheat grains under the different treatments. Salinity stress increased total amino acids and non-essential amino acids; in contrast, it decreased essential amino acids and the ratio of essential/non-essential amino acids of wheat grains as compared with the control plants (Table 5). In the present study, salinity stress caused an obvious alteration in essential amino acids. Leucine, phenylalanine, and threonine increased in SNP- or arginine-treated plants. On the contrary, isoleucine, lysine, and valine decreased due to salinity stress. Both arginine and SNP treatments improved almost all essential amino acid contents in wheat grains, except for methionine, which was decreased. Furthermore, salinity stress led to a decrease in non-essential amino acids—cysteine, alanine, aspartic acid, glutamic acid, arginine, and serine contents—and a great increase in proline and the amino acid glycine. Moreover, salinity stress increased proline contents, with further upregulation found as a result of the applied protectants. 

Different treatments of arginine or SNP increased the total essential amino acid, total non-essential amino acid, and total amino acid contents of wheat grains either under normal conditions or stressed conditions. Furthermore, the ratio of essential amino acids/non-essential amino acids increased with arginine or SNP treatment under normal conditions. However, arginine or SNP treatment under stressed conditions decreased the ratio compared with its corresponding controls. Both arginine and SNP treatments caused stimulation in the glycine content whether under the control or salt stress conditions. The glutamic acid content decreased under salinity stress compared to the control, but the treatment with arginine or SNP increased the glutamic acid content under both normal and salinity stress conditions.

## 3. Discussion

Environmental factors, especially salinity stress, drastically influence the growth, metabolism, yield, and composition of wheat grains. Treatments with NO donors to reduce the unfavorable consequences of salinity stress on wheat plants were studied. 

In the current study, salt stress significantly reduced the various growth indices of wheat plants (Table 1), similar to the findings reported for wheat, Indian mustard, and okra plants [7,21,22]. The decrease in growth indices might be due to the reduction in cell division, cell expansion, and inhibition of apical growth [23]. Furthermore, salinity stress limits CO_2_ fixation via stomatal closure, thus disturbing the normal electron flow of carbon reduction in the Calvin cycle, which is associated with low fresh and dry matter production [24,25]. These harmful effects were counteracted by external NO treatment (Table 1).

The analyzed data show that foliar application of arginine or SNP could ameliorate the negative effect of salinity stress on wheat growth traits. The data are congruent with those obtained in sunflower seed after priming in arginine or SNP, which enhanced the growth parameters of the plants under salinity stress [26,27] by maintaining ion homeostasis, re-establishing the redox balance, and thus enhancing growth [28]. In addition, NO plays a role in regulating plant metabolism [29,30], photosynthetic pigments [31], ameliorating oxidative stress [32,33,34], and plant hormones [35,36,37]. In contrast, the role of arginine in increasing growth parameters might be attributed to the increase in polyamine biosynthesis, which might be involved in a wide range of biological processes including growth [38]. The data of the present study also add that the promotive effect of external treatment using SNP or its precursor arginine on wheat plant salinity tolerance might be attributed to the enhancement of the leaf water content and photosynthetic pigments and accumulation of osmoprotectant compounds and an antioxidative defense system. The NO molecule permeates membranes and serves as a key signaling molecule in plants [39].

A common symptom of salinity stress is the destruction of photosynthetic pigments, which causes impaired photosynthesis [40,41] and thus growth retardation, as revealed by the current study. These results are substantiated by earlier studies on *Lupines termis* [42], chickpea [43], and okra [26]. This reduction in photosynthetic pigments might be due to the increased Na^+^ and Cl^−^ ions, which, in turn, reduce chlorophyll biosynthesis by affecting the activity of Fe^+3^, which contains chlorophyll-producing enzymes [44]. In addition, salinity stress may cause an increase in chlorophyllase activity, the accumulation of ROS [45], and disorders of pigment–protein complexes [46]. However, the carotenoid content increases under salinity stress. It has been reported that carotenoids are involved in regulating photoprotection against photo-oxidation [47]. Moreover, during salt stress, the synthesis of carotenoids increases due to the antioxidant properties of these compounds. This helped to reduce the oxidative damage caused by salinity stress in chickpea [48]. In contrast, these reduced effects of salt stress on total pigments, chl ***a***, and chl ***b*** were counteracted by exogenous treatment of arginine or SNP. This could be ascribed to the significant function of NO in scavenging ROS, reducing oxidative damage in the photosynthetic apparatus, increasing the chlorophyll concentration [49], and protecting cell organelles carrying chlorophyll from salt-induced toxicity [50]. In agreement with [31], the application of arginine or SNP enhanced the chlorophyll content of sunflower plants exposed to salt stress. The positive role of arginine or SNP could be attributed to enhancing the iron content of plants, which results in better retention of chlorophyll under stressful conditions [51]. 

In the present work, the attenuation of the IAA content by salinity stress in wheat was alleviated by the external treatment of arginine or SNP at different concentrations (Figure 2). The reduction in IAA might be the result of the increases in IAA destruction via the enhancement of IAA oxidase activity [52]. The increase in IAA boosted the growth rate due to its role in enhancing cell division and/or cell enlargement [53]. These findings are supported by previous studies [31,54]. Furthermore, the phenolic contents increased due to salinity stress. Phenolics are antioxidant compounds that contribute to the defense system and scavenge ROS induced by different stresses [55]. The activation of phenolics was further upregulated by exogenous treatment using NO sources. Osmotic adjustment in plant cells takes place with increases in contents of osmoprotectant compounds such as TSS, proline [56], and amino acids [1]. Thus, to resist osmotic stress, plants under salinity stress accumulate both soluble proteins and carbohydrates as a cellular defense against oxidative damage via lipid peroxidation inhibition and free radical scavenging [48]. In the current study, wheat plants under saline conditions expressed a significant increase in free amino acids, proline, and TSS (Table 2). The inducing role of salinity stress on osmoprotectants was confirmed earlier in okra, soybean, and rice plants [26,57,58]. Proline is a non-toxic osmotic solute that stabilizes the macromolecule and organelle structure [59]. Under different environmental stresses, proline performs multiple functions in plant cells such as adjusting the pressure potential and membrane integrity, scavenging ROS, and improving growth [60,61]. The results of earlier studies of arginine [14] are in harmony with our results supporting the promotive role of NO, polyamines, or proline under stress conditions. Our results agree with the findings reported for chickpea [62], *Pinus eldarica* [63], and sunflower [31]. 

In the present study, salinity stress induced high oxidative damage in wheat leaves, as denoted by the significant increase in H_2_O_2_, which was reflected in the induction of the MDA content. However, the prevention of oxidative burst mediated by NO could be ascribed to enhancing the elasticity and stability of cells via strengthening of a phospholipid bilayer, and to stimulating membrane fluidity and adjusting ROS [64]. NO as a free gas can act as a chain-disintegrating agent during the reaction of lipid peroxidation via cooperation with lipid alkoxyl and peroxyl radicals [65]. The current findings also reveal that the lower chlorophyll content in wheat plants subjected to salt stress is linked to an excess of H_2_O_2_ in the leaves. These data imply that NO may be important in reducing the adverse effects of salt stress on chlorophyll, possibly by reducing H_2_O_2_ formation. Another possible function of NO-induced salinity tolerance in wheat plants might be to boost antioxidative defense mechanisms to scavenge H_2_O_2_, resulting in a higher chlorophyll content.

The state of the cellular ROS balance is mediated by the implication of enzymatic antioxidants that combat oxidative damage. The salinized plants exhibited enhanced levels of CAT and POX activities that could be involved in quenching ROS from stressed cells, limiting cellular damage, and improving plants’ oxidative capacity to defend against stress [1]. Moreover, increases in SOD, POX, and CAT activities enhance the elimination of excess ROS molecules, conferring higher stress resistance on wheat plants. The promotive effect of either arginine or SNP on antioxidant enzymes of wheat plants might be due to the protective effect of the released NO [30]. Moreover, NO serves as a signaling molecule that enhances salt tolerance via increasing various antioxidant enzyme production rates in the mitochondria [20]. In this sense, NO prevents oxidative damage in stressed plants by regulating redox homeostasis and boosting the activity of H_2_O_2_-scavenging enzymes [66]. Thus, exogenous NO sources could induce endogenous NO production, which has signaling or scavenger properties even long after the NO donor is diminished. Moreover, NO triggers the expression of antioxidant genes producing higher enzyme activities, possibly by post-translational alterations that protect the plants against stress [67,68]. 

It is worth mentioning that NR is the main pathway of NO production. In the present study, salinity stress reduced the foliar activity of NR similar to that reported by [69,70]. NR activity was triggered by NO application, which might be reflected in the endogenous NO level. Thus, the stimulation of NR activity was a consequence of enhanced NO production, hence inducing various metabolic regulations and antioxidative properties. Thus, NR activation might mediate stress conditions by inducing endogenous NO that can, in turn, induce a set of stress ameliorative events even after the consumption of NO donors.

As an insight into the end of the life cycle of wheat plants under salinity stress, yield and its components were analyzed. As a result of prolonged salinity stress and metabolic disorders, significant decreases in all yield traits in combination with different growth parameters measured at harvest were observed. Furthermore, salinity also decreased the carbohydrate contents of the wheat. These reductions in wheat yields were concomitant with a major reduction in growth indices (Table 1) and photosynthetic pigments (Figure 1). In turn, less accumulation of photosynthetic outputs (carbohydrates) is needed for grain filling or a reduction in the rate of transport of carbohydrates from leaves to developing seeds [71]. The change in grains’ carbohydrate content is very crucial due to their direct relationship with other physiological processes such as respiration [72]. The reduction in yield is associated with delayed flowering and decreasing flower number and pod set [73]. Different NO sources significantly increased yield and its components as well as carbohydrate contents either under normal conditions or salinity stress conditions. These results are in harmony with those recorded by [74] in various wheat cultivars using different SNP levels (0.1 and 0.2 mM). 

The general metabolism of plants or grains is altered towards energy conservation and stress defense, which causes a delay in the growth and development of grains. Plant amino acid components are involved directly or indirectly in controlling plant responses to environmental signals linked to abiotic or biotic stress, especially osmotic adjustment [75]. Concomitantly, a higher level of different amino acids is associated with an osmotic adjustment which results in free radical scavenging and protein integrity [76]. The accumulation of most of the individual amino acids including those of the glutamate pathway: glutamate, proline, arginine, histidine, and ornithine, increased in the presence of salt stress [77]. From the free amino acid profile tested, proline—which was found to be increased in salt-treated plants—acts as an osmoprotectant molecule and has been shown to increase in response to salinity stress [78]. However, salinized plants mainly increase the proline content at the expense of glutamate. Under such circumstances, glutamate’s conversion to proline might occur because of a shift in plant priorities towards better survival [79]. Furthermore, the increase in the isoleucine content may be ascribed to the accumulation of proline. The current results agree with those of [80], in that salinity tolerance is related to some amino acids, e.g., proline, glutamate, ornithine, and aspartate. In contrast, non-essential amino acids alanine, aspartic acid, and serine contents were reduced by salinity. However, the significant increase in glycine and cysteine contents in salinity-stressed plants could be a result of the osmotic adjustment. Of the amino acids related to the maintenance of cellular metabolism, increases in the levels of tryptophan, phenylalanine, tyrosine, and histidine are likely related to defensive responses in soybeans under stress [81]. The role of different amino acids is implicated in various regulatory roles in plants [10]: for example, arginine and methionine are precursors of polyamines and ethylene, respectively. The aromatic amino acids phenylalanine, tyrosine, and tryptophan are intermediates or central molecules for producing secondary metabolites with varying biological importance and various health-promoting criteria [10]. Thus, it seems that NO application upregulates the nutritional status of the produced grains of wheat plants under the tested conditions. Therefore, there is a need for further studies on the genes affecting amino acid homeostasis in grains, leaves, and roots and their interaction. 

## 4. Materials and Methods

### 4.1. Experimental Design

A pot experiment was conducted from November to April of 2018/2019 at the greenhouse of the National Research Centre, Dokki, Cairo, Egypt. The experiment aimed to study the effect of foliar application of different concentrations of SNP and the amino acid arginine on wheat plants grown under salinity stress conditions. 

Plastic pots were filled with 7 kg clay soil, which was mixed with yellow sand in a proportion of 3:1 (v:v) to reduce compaction and improve drainage. Then, 1.06 g of granular ammonium sulfate (20.5% N) and single superphosphate (15% P_2_O_5_) was added to each pot. The N and P fertilizers were mixed thoroughly with the soil in each pot before sowing. The field water capacity was 0.36. The pots were divided into two main groups: a control group irrigated with tap water (S_0_ =1.2 mM NaCl equal to an EC of 0.03 dSm^−1^) and a salinity group irrigated with saline water (S_1_ = 85.5 mM NaCl equal to an EC of 5.10 dSm^−1^), according to Stroganov (1962) [82]. 

Uniform wheat grains (*Triticum aestivum* L. cv. Benisuif 4) were surface-sterilized with 1% sodium hypochlorite solution for 2 min and then thoroughly washed with distilled water several times. Air-dried sterilized grains were sown at 30 mm depth in the previously prepared pots. Ten days after sowing, the seedlings were thinned to 5 seedlings per pot.

Each group was subdivided into five subgroups; one of them was sprayed with distilled water (control), and the other four subgroups were foliar sprayed with two concentrations of SNP (2.5 and 5.0 mM) and two concentrations of arginine (10 and 20 mM). The SNP and arginine used in the present study were supplied by Sigma-Aldrich. A preliminary germination experiment using different concentrations of SNP (0.0, 1.25, 2.5, 5.0, 7.5, and 10.0 mM) and arginine (0.0, 5.0, 10.0, 15.0, 20.0, 25.0 and 30.0 mM) was conducted. Then, the appropriate concentrations of SNP and arginine were chosen based on the results of growth characteristics. SNP and arginine at the calculated concentrations were dissolved in distilled water and freshly used. Each treatment consisted of three replicates distributed in a completely randomized design system. Spraying of wheat plants with SNP or arginine was performed twice (20 mL plant^−1^) on the 45th and the 60th days after sowing. Plant samples were taken 75 days after sowing for morphological and biochemical analyses. 

### 4.2. Growth Characteristics and Yield Components Traits

Growth characteristics are presented in shoot length (cm), leaves number/tiller, flag leaves area (cm^2^), root length (cm), tillers fresh and dry weight (g) and root fresh and dry weight (g). The samples were dried at 80 °C for 2–4 days until they achieved constant dry weight (DW) for determination. At harvest, the shoot length (cm) and weight (g), spike length (cm), weight/plant (g), grains weight/plant (g), and 1000 grains weight (g) were measured. 

### 4.3. Biochemical Analysis

Plant samples were frozen in liquid nitrogen and then kept in a deep freezer for chemical analysis.

Photosynthetic pigments: Chlorophyll a, chlorophyll b and carotenoids in fresh leaves were determined according to the method described by Lichtenthaler and Buschmann (2001) [83] using a spectrophotometer (Shimadzu UV−1700, Tokyo, Japan). The values of photosynthetic pigments were expressed in μg g^−1^ FW.

Endogenous phytohormones: Indole acetic acid (IAA) was extracted and analyzed by Larsen et al. [84] A known weight of the fresh samples was taken and extracted with 85% cold methanol (*v*/*v*) three times at 0 °C. The combined extracts were collected and made up to a known volume with cold methanol. Then, 1 mL of the methanolic extract and 4 mL of PDAB reagent (para-dimethylamino benzoic acid 1 g dissolved in 50 mL HCl, 50 mL of ethanol 95%) were taken and left for 60 min at 30–40 °C. The developing color was spectrophotometrically measured at a wavelength of 530 nm. 

Non-enzymatic antioxidants: Phenolic compounds in leaves were extracted as IAA and determined using a spectrophotometer as described by Danil et al. [85]. Next, 0.5 mL of the extract was added to 0.5 mL Folin–Ciocalteu reagent, shaken and allowed to stand for 3 min. Then, one ml of saturated sodium carbonate was added followed by distilled water shaken and allowed to stand for 60 min. 

The compatible solutes: Free amino acids in leaves were extracted according to Kalsoom et al., (2016) [86]. Free amino acids were estimated with the ninhydrin reagent method according to Sorrequieta et al. [87]. Proline was extracted as free amino acid and assayed according to Bates et al. [88]. 

Total soluble sugars (TSS) were extracted by overnight submersion of dry tissue in 10 mL of 80% (*v*/*v*) ethanol at 25 °C with periodic shaking and centrifuged at 600 g. The supernatant was evaporated till completely dried then dissolved in a known volume of distilled water to be ready for the determination by the method of Chow and Landhausser [89]. 

The determination of the total carbohydrates of the yielded grains was carried out according to Albalasmeh et al. [90]. First, 0.5 g of dried tissue was placed in a test tube, and then 10 mL of sulfuric acid (1N) was added. The tube was sealed and placed overnight in an oven at 100 °C. Then, 1 mL of the filtered solution was transferred into the test tube and treated with 1 mL of 5% aqueous phenol solution followed by 5.0 mL of concentrated sulfuric acid. The tubes were thoroughly shaken for ten minutes and then placed in a water bath at 23–30 °C for 20 min. The total sugars were measured at 490 nm using Shimadzu spectrophotometer model UV 1201.

Reactive oxygen species and membrane damage: Hydrogen peroxide level was determined in leaves colorimetrically using the method of Yu et al. [91]. The level of lipid peroxidation was measured by determining the levels of malondialdehyde (MDA) content based on the method of Hodges et al. [92]. 

Enzymatic antioxidants: Enzyme extracts were collected according to the method described by Chen et al. [93]. Leaf tissues were homogenized in ice-cold phosphate buffer (50 mM, pH 7.8), followed by centrifugation at 8000 rpm and 4 °C for 15 min. The supernatant was used immediately to determine the activities of enzymes. Catalase (CAT, EC 1.11.1.6) was calculated by nitro-blue-tetrazolium (NBT) reduction method [93]. The enzyme activities were calculated by Kong et al. [94]. Superoxide dismutase (SOD, EC 1.12.1.1) activity was spectrophotometrically assayed at 560 nm by the NBT reduction method according to Chen et al. [93]. Peroxidase (POX, EC 1.11.1.7) activity was assayed according to the method of Kumar and Khan [95].

The activity of nitrate reductase (NR, EC 1.7. 1. 1): Nitrate reductase (NR) was extracted as described by Foyer et al., (1998) [96]. NR was measured according to Jaworski (1971) [97]. Amino acids profile; the identification and determination of the amino acid composition of wheat grains were carried out by using HPLC (Eppendorf, Germany) according to Gehrke [98].

### 4.4. Statistical Analysis

The data were analyzed by a one-way ANOVA test using the SPSS 21.0 software program. All statistical differences are presented compared to the untreated control. Means were recorded for three replicate values. Means were compared using Duncan’s multiple range test, and statistical significance was evaluated at (*p* ≤ 0.05) [99].

## 5. Conclusions

In brief, the main mechanism of SNP and arginine in alleviating the damaging impact of salinity stress is the induction of the endogenous foliar content of NR activities, which may upregulate the endogenous NO, thereby mediating multilayered tolerance mechanisms. Thus, SNP or arginine modulated photosynthetic pigments, ameliorated oxidative stress disorders, accumulated osmoprotectants, enhanced IAA contents and phenolic biosynthesis, and triggered superoxide radical- and hydrogen-peroxide-scavenging enzymes. All these alterations positively correlated with the growth, and hence the yield, of the wheat plants under salinity stress. The individual amino acid and carbohydrate contents of grains were elevated by the NO sources, implying the ability of NO to improve the nutritional status of wheat grains.

## Figures and Tables

**Table 1:** Effect of arginine or SNP on growth indices of wheat plants under salinity stress. S_0_ = 1.2 mM NaCl, S_1_ = 85.5 mM NaCl.

Salinity	Treatments	Shoot Length (cm)	Leaves No/tiller	Flag Leaf Area (cm^2^)	Tillers Fresh Weight (g)	Tillers Dry Weight (g)	Root Length (cm)	Root Fresh WT (g)	Root Dry WT (g)
**S_0_**	**Control**	56.3 ^c^ ± 1.45	4.7 ^bc^ ± 0.33	30.5 ^b^ ± 0.14	5.6 ^c^ ± 0.12	1.8 ^b^ ± 0.05	13.7 ^b^ ± 0.33	1.8 ^b^ ± 0.05	0.92 ^b^ ± 0.02
	**Arg 10 mM**	63.3 ^d^ ± 0.88	5.3 ^bc^ ± 0.33	36.9 ^de^ ± 0.96	6.6 ^e^ ± 0.15	2.1 ^cd^ ± 0.06	16.3 ^de^ ± 0.33	2.1 ^cd^ ± 0.06	1.10 ^d^ ± 0.02
	**Arg 20 mM**	70.3 ^e^ ± 1.20	5.7 ^bc^ ± 0.33	42.6 ^f^ ± 0.79	8.1 ^f^ ± 0.20	2.7 ^e^ ± 0.03	17.3 ^e^ ± 0.88	2.7 ^e^ ± 0.03	1.28 ^e^ ± 0.00
	**SNP 2.5 mM**	63.0 ^d^ ± 0.57	5.7 ^bc^ ± 0.33	35.4 ^d^ ± 0.26	6.1 ^d^ ± 0.09	2.3 ^d^ ± 0.04	16.7 ^de^ ± 0.33	2.3 ^d^ ± 0.04	1.12 ^d^ ± 0.031
	**SNP 5.0 mM**	70.0 ^e^ ± 1.00	6.0 ^c^ ± 0.00	42.2 ^f^ ± 0.41	8.3 ^f^ ± 0.11	2.8 ^e^ ± 0.05	20.0 ^f^ ± 0.57	2.8 ^e^ ± 0.05	1.26 ^e^ ± 0.01
**S_1_**	**Control**	43.7 ^a^ ± 0.67	4.3 ^a^ ± 0.33	24.2 ^a^ ± 0.38	4.1 ^a^ ± 0.05	1.1 ^a^ ± 0.06	11.7 ^a^ ± 0.33	1.1 ^a^ ± 0.06	0.84 ^a^ ± 0.02
	**Arg 10 mM**	51.7 ^b^ ± 0.33	4.7 ^ab^ ± 0.33	31.8 ^bc^ ± 0.38	5.2 ^b^ ± 0.04	1.7 ^b^ ± 0.023	13.3 ^b^ ± 0.33	1.7 ^b^ ± 0.023	0.95 ^bc^ ± 0.018
	**Arg 20 mM**	59.0 ^c^ ± 1.00	5.0 ^ab^ ± 0.00	37.5 ^e^ ± 0.93	5.8 ^c^ ± 0.08	2.0 ^c^ ± 0.052	15.3 ^cd^ ± 0.66	2.0 ^c^ ± 0.052	1.03 ^c^ ± 0.03
	**SNP 2.5 mM**	51.0 ^b^ ± 1.15	5.3 ^bc^ ± 0.33	32.9 ^c^ ± 0.27	4.9 ^b^ ± 0.10	1.8 ^b^ ± 0.06	14.0 ^bc^ ± 0.57	1.8 ^b^ ± 0.06	0.97 ^bc^ ± 0.03
	**SNP 5.0 mM**	58.7 ^c^ ± 0.33	5.3 ^bc^ ± 0.33	38.5 ^e^ ±0.66	5.8 ^c^ ± 0.0	2.1 ^cd^ ± 0.04	16.3 ^de^ ± 0.33	2.1 ^cd^ ± 0.04	1.12 ^d^ ± 0.03

All the statistical differences were presented relative to the untreated control. Data are means of three replicates ± SE; means with different letters within the same column are significantly different (*p* ≤ 0.05).

**Table 2:** Effect of arginine or SNP on free amino acids, proline (μg g^−1^ fresh weight), and total soluble sugars (TSS) (mg g^−1^ dry weight) of wheat plants grown under salinity stress. S_0_ = 1.2 mM NaCl, S_1_ = 85.5 mM NaCl.

Salinity	Treatments	Free Amino Acids	Proline	TSS
**S_0_**	**Control**	137.15 ^a^ ± 0.86	23.83 ^a^ ± 0.10	21.55 ^a^ ± 0.11
	**Arg 10 mM**	152.84 ^c^ ± 0.28	36.25 ^c^ ± 0.23	23.44 ^b^ ± 0.05
	**Arg 20 mM**	160.15 ^d^ ± 1.44	49.22 ^e^ ± 0.2 4	27.30 ^d^ ± 0.19
	**SNP 2.5 mM**	147.92 ^b^ ± 0.92	33.93 ^b^ ± 0.13	25.66 ^c^ ± 0.008
	**SNP 5.0 mM**	154.83 ^c^ ± 1.43	42.43 ^d^ ± 0.62	28.90 ^e^ ± 0.14
**S_1_**	**Control**	152.59 ^c^ ± 0.14	37.25 ^c^ ± 0.34	25.56 ^c^ ± 0.049
	**Arg 10 mM**	168.59 ^f^ ± 0.60	51.13 ^f^ ± 0.10	31.18 ^f^ ± 0.19
	**Arg 20 mM**	176.15 ^g^ ± 1.44	54.25 ^g^ ± 0.34	36.31 ^h^ ± 0.36
	**SNP 2.5 mM**	163.93 ^e^ ± 0.33	54.75 ^g^ ± 0.63	33.75 ^g^ ± 0.05
	**SNP 5.0 mM**	171.08 ^f^ ± 0.82	68.08 ^h^ ± 0.32	37.30 ^i^ ± 0.37

All the statistical differences were presented relative to the untreated control. Data are means of three replicates ± SE; means with different letters within the same column are significantly different (*p* ≤ 0.05).

**Table 3:** Effect of arginine on MDA, H_2_O_2_ (μg g^−1^ fresh weight) and antioxidant enzymes (CAT, SOD, POX (μg g^−1^ fresh weight/h) and NR (n mole NO_2_/g fresh weight/h) of wheat plant grown under salinity stress. S_0_ = 1.2 mM NaCl, S_1_ = 85.5 mM NaCl.

Salinity	Treatments	MDA	H_2_O_2_	CAT	SOD	POX	NR
**S_0_**	**Control**	7.66 ^d^ ± 0.00	5.24 ^c^ ± 0.05	55.83 ^a^ ± 0.28	38.18 ^a^ ± 0.28	11.75 ^a^ ± 0.057	320.76 ^d^ ± 0.25
	**Arg 10 mM**	6.58 ^c^ ± 0.05	4.23 ^b^ ± 0.04	58.16 ^bc^ ± 0.85	43.95 ^b^ ± 0.17	12.97 ^bc^ ± 0.31	328.50 ^fg^ ± 0.66
	**Arg 20 mM**	6.34 ^bc^ ± 0.07	3.73 ^a^ ± 0.22	59.15 ^cd^ ± 0.28	46.25 ^c^ ± 0.23	13.15 ^bc^ ± 0.28	331.52 ^g^ ± 0.57
	**SNP 2.5 mM**	6.08 ^ab^ ± 0.03	4.29 ^b^ ± 0.13	57.40 ^b^ ± 0.14	45.60 ^c^ ± 0.01	12.53 ^ab^ ± 0.06	321.52 ^de^ ± 0.00
	**SNP 5.0 mM**	5.93 ^a^ ± 0.04	3.53 ^a^ ± 0.06	59.65 ^d^ ± 0.00	47.30 ^d^ ± 0.20	13.70 ^c^ ± 0.02	329.95 ^g^ ± 0.17
**S_1_**	**Control**	13.45 ^h^ ± 0.11	9.23 ^e^ ± 0.06	63.69 ^e^ ± 0.02	51.38 ^e^ ± 0.02	20.93 ^d^ ± 0.33	296.83 ^a^ ± 0.29
	**Arg 10 mM**	11.6 ^g^ ± 0.14	6.53 ^d^ ± 0.06	70.45 ^f^ ± 0.46	60.58 ^f^ ± 0.53	32.23 ^e^ ± 0.41	306.38 ^b^ ± 3.54
	**Arg 20 mM**	8.40 ^e^ ± 0.14	5.38 ^c^ ± 0.03	77.25 ^h^ ± 0.80	72.15 ^h^ ± 0.28	40.58 ^g^ ± 0.53	318.08 ^d^ ± 1.40
	**SNP 2.5 mM**	10.44 ^f^ ± 0.11	6.28 ^d^ ± 0.07	73.58 ^g^ ± 0.037	62.88 ^g^ ± 0.21	34.08 ^f^ ± 0.25	314.08 ^c^ ± 0.90
	**SNP 5.0 mM**	7.65 ^d^ ± 0.17	5.07 ^c^ ± 0.14	81.27 ^i^ ± 0.14	75.00 ^i^ ± 0.20	46.25 ^h^ ± 0.23	325.15 ^ef^ ± 0.28

All the statistical differences were presented relative to the untreated control. Data are means of three replicates ± SE; means with different letters within the same column are significantly different (*p* ≤ 0.05).

**Table 4:** Effect of arginine or SNP on yield, its components, and total carbohydrate% of wheat plant grown under salinity stress. S_0_ = 1.2 mM NaCl, S_1_ = 85.5 mM NaCl.

Salinity	Treatments	Shoot Length (cm)	Spike Length (cm)	Spike Weight (g)	Shoot Weight (cm)	Grain Weight (g)	1000 Kernel Weight (g)	Total Carbohydrate (%)
**S_0_**	**Control**	63 ^bc^ ± 0.33	9.33 ^b^ ± 0.33	2.63 ^c^ ± 0.14	2.18 ^b^ ± 0.04	1.80 ^bc^ ± 0.06	35.38 ^c^ ± 0.37	45.83 ^d^ ± 0.11
	**Arg 10 mM**	70 ^d^ ± 1.00	11.66 ^c^ ± 0.33	3.50 ^e^ ± 0.05	2.59 ^c^ ± 0.01	2.22 ^d^ ± 0.035	38.50 ^d^ ± 0.19	46.90 ^f^ ± 0.028
	**Arg 20 mM**	79 ^e^ ± 0.88	13.33 ^d^ ± 0.33	3.88 ^f^ ± 0.07	2.64 ^c^ ± 0.03	2.43 ^e^ ± 0.025	42.97 ^e^ ± 0.79	48.80 ^g^ ± 0.08
	**SNP 2.5 mM**	76 ^e^ ± 0.66	14.00 ^d^ ± 0.57	3.20 ^d^ ± 0.04	2.45 ^c^ ± 0.01	2.28 ^de^ ± 0.06	39.36 ^d^ ± 0.20	46.98 ^f^ ± 0.02
	**SNP 5.0 mM**	76 ^e^ ± 2.72	14.33 ^d^ ± 0.33	4.05 ^f^ ± 0.10	2.90 ^d^ ± 0.14	2.61 ^f^ ± 0.14	42.57 ^e^ ± 0.49	48.88 ^g^ ± 0.13
**S_1_**	**Control**	52 ^a^ ± 1.00	8.00 ^a^ ± 0.57	1.97 ^a^ ± 0.07	1.90 ^a^ ± 0.03	1.16 ^a^ ± 0.03	29.85 ^a^ ± 0.62	43.45 ^a^ ± 0.11
	**Arg 10 mM**	63 ^bc^ ± 0.88	9.66 ^b^ ± 0.33	2.33 ^b^ ± 0.04	2.19 ^b^ ± 0.02	1.75 ^b^ ± 0.03	32.30 ^b^ ± 0.39	44.90 ^b^ ± 0.13
	**Arg 20 mM**	66 ^cd^ ± 1.20	11.66 ^c^ ± 0.33	2.65 ^c^ ± 0.05	2.45 ^c^ ± 0.11	1.93 ^c^ ± 0.02	34.87 ^c^ ± 0.21	45.90 ^d^ ± 0.13
	**SNP 2.5 mM**	62 ^b^ ± 0.66	9.66 ^b^ ± 0.33	2.19 ^b^ ± 0.05	2.25 ^b^ ± 0.01	1.25 ^a^ ± 0.01	33.32 ^b^ ± 0.24	45.18 ^c^ ± 0.03
	**SNP 5.0 mM**	68 ^d^ ± 0.66	11.66 ^c^ ± 0.33	2.35 ^b^ ± 0.005	2.52 ^c^ ± 0.03	1.69 ^b^ ± 0.029	34.65 ^c^ ± 0.27	46.23 ^e^ ± 0.06

All the statistical differences were presented relative to the untreated control. Data are means of three replicates ± SE; means with different letters within the same column are significantly different (*p* ≤ 0.05).

**Table 5:** Effect of arginine (20 mM) or SNP (5.0 mM) on individual amino acids composition (mg 100 g^−1^ DW) of the wheat grains grown under salinity stress. S_0_ = 1.2 mM NaCl, S_1_ = 85.5 mM NaCl.

Treatments	S_0_			S_1_		
	Control	Arg 20 mM	SNP 5.0 mM	Control	Arg 20 mM	SNP 5.0 mM
**Cysteine**	14.73 ^a^ ± 0.18	19.25 ^c^ ± 0.23	19.68 ^c^ ± 0.18	17.52 ^b^ ± 0.20	20.85 ^d^ ± 0.24	24.65 ^e^ ± 0.23
**Methionine**	1.49 ^b^ ± 0.09	1.35 ^ab^ ± 0.06	1.75 ^c^ ± 0.07	1.81 ^c^ ± 0.086	1.23 ^a^ ± 0.46	1.35 ^ab^ ± 0.04
**Total sulfur AA**	16.22 ^a^ ± 0.11	20.60 ^c^ ± 0.14	21.43 ^d^ ± 0.12	19.33 ^b^ ± 0.17	22.08 ^e^ ± 0.23	26.00 ^f^ ± 0.19
**Phenylalanine**	4.39 ^a^ ± 0.017	6.68 ^c^ ± 0.075	6.27 ^b^ ± 0.75	7.65 ^d^ ± 0.086	8.95 ^f^ ± 0.11	8.35 ^e^ ± 0.09
**Tyrosine**	6.79 ^a^ ± 0.08	8.75 ^c^ ± 0.04	8.95 ^c^ ± 0.07	7.75 ^b^ ± 0.05	9.45 ^d^ ± 0.07	9.68 ^e^ ± 0.03
**Histidine**	9.52 ^a^ ± 0.05	13.78 ^c^ ± 0.07	14.65 ^d^ ± 0.11	9.65 ^a^ ± 0.08	11.68 ^b^ ± 0.06	11.65 ^b^ ± 0.03
**Tryptophan**	2.32 ^b^ ± 0.01	2.51 ^c^ ± 0.03	1.65 ^a^ ± 0.01	3.12 ^d^ ± 0.06	3.32 ^e^ ± 0.04	4.34 ^f^ ± 0.02
**Total aromatic AA**	23.02 ^a^ ± 0.17	31.72 ^c^ ± 0.23	31.52 ^c^ ± 0.26	28.17 ^b^ ± 0.20	33.40 ^d^ ± 0.26	34.02 ^d^ ± 0.36
**Threonine**	8.27 ^a^ ± 0.07	13.49 ^d^ ± 0.11	13.68 ^d^ ± 0.11	9.65 ^b^ ± 0.08	9.68 ^b^ ± 0.07	11.52 ^c^ ± 0.17
**Valine**	9.79 ^a^ ± 0.09	12.62 ^c^ ± 0.12	13.84 ^d^ ± 0.06	9.64 ^a^ ± 0.02	11.68 ^b^ ± 0.08	13.85 ^d^ ± 0.24
**Leucine**	8.46 ^a^ ± 0.02	16.75 ^d^ ± 0.08	17.65 ^e^ ± 0.07	8.68 ^a^ ± 0.02	9.68 ^b^ ± 0.13	9.95 ^c^ ± 0.02
**Isoleucine**	31.74 ^c^ ± 0.19	37.95 ^d^ ± 0.23	38.64 ^e^ ± 0.12	23.68 ^a^ ± 0.13	29.68 ^b^ ± 0.05	31.52 ^c^ ± 0.07
**Lysine**	32.34 ^c^ ± 0.12	45.25 ^e^ ± 0.13	46.95 ^f^ ± 0.17	27.75 ^a^ ± 0.05	31.52 ^b^ ± 0.11	33.52 ^d^ ± 0.11
**Essential AA**	106.00 ^b^ ± 0.42	147.87 ^e^ ± 0.28	153.43 ^f^ ± 0.31	98.51 ^a^ ± 0.21	114.10 ^c^ ± 0.34	121.71 ^d^ ± 0.17
**Aspartic acid**	13.18 ^c^ ± 0.17	11.85 ^a^ ± 0.12	14.65 ^d^ ± 0.06	12.65 ^b^ ± 0.08	15.85 ^e^ ± 0.13	16.38 ^f^ ± 0.08
**Serine**	4.63 ^b^ ± 0.06	8.13 ^d^ ± 0.12	7.68 ^c^ ± 0.15	3.68 ^a^ ± 0.19	4.84 ^b^ ± 0.08	4.69 ^b^ ± 0.17
**Glutamic acid**	37.87 ^c^ ± 0.29	43.49 ^e^ ± 0.17	43.65 ^e^ ± 0.35	32.68 ^a^ ± 0.11	35.68 ^b^ ± 0.12	39.68 ^d^ ± 0.28
**Proline**	40.26 ^a^ ± 0.25	51.63 ^c^ ± 0.29	45.65 ^b^ ± 0.36	59.68 ^d^ ± 0.17	85.65 ^e^ ± 0.28	96.35 ^f^ ± 0.52
**Glycine**	36.52 ^a^ ± 0.14	46.59 ^c^ ± 0.23	47.65 ^d^ ± 0.28	45.62 ^b^ ± 0.23	59.68 ^e^ ± 0.34	59.68 ^e^ ± 0.38
**Alanine**	14.52 ^d^ ± 0.17	13.85 ^c^ ± 0.07	15.84 ^e^ ± 0.28	8.65 ^a^ ± 0.11	11.68 ^b^ ± 0.18	11.68 ^b^ ± 0.28
**NH_4_**	13.72 ^e^ ± 0.29	8.24 ^b^ ± 0.07	6.65 ^a^ ± 0.08	13.85 ^e^ ± 0.08	11.65 ^c^ ± 0.20	12.65 ^d^ ± 0.11
**Arginine**	28.35 ^b^ ± 0.17	32.52 ^d^ ± 0.14	35.68 ^e^ ± 0.07	23.68 ^a^ ± 0.12	32.68 ^d^ ± 0.28	29.85 ^c^ ± 0.06
**Non-essential AA**	184.54 ^a^ ± 0.70	214.29 ^c^ ± 1.1	212.05 ^c^ ± 0.75	205.20 ^b^ ± 0.63	258.65 ^d^ ± 1.2	279.78 ^e^ ± 1.4
**Total amino acids**	318.89 ^a^ ± 1.2	394.68 ^c^ ± 0.86	401.16 ^d^ ± 1.6	327.39 ^b^ ± 0.66	405.43 ^e^ ± 1.1	431.34 ^f^ ± 1.5
**Ess/Non Ess**	0.574 ^b^ ± 0.02	0.690 ^c^ ± 0.02	0.724 ^c^ ± 0.01	0.480 ^a^ ± 0.01	0.441 ^a^ ± 0.01	0.435 ^a^ ± 0.01

All the statistical differences were presented relative to the untreated control. Data are means of three replicates ± SE; means with different letters within the same column are significantly different (*p* ≤ 0.05).

## Data Availability

Not applicable.
